# Supplementary material for: A UMLS-based spell checker for natural language processing in vaccine safety
Source: BMC Med Inform Decis Mak. 2007 Feb 12;7:3. doi: 10.1186/1472-6947-7-3 (PMC1805499; doi:10.1186/1472-6947-7-3)
Supplement: Additional file 13 — RAP application directory. Sets up directory for RAP (RDF application for PHP) source code [file 1472-6947-7-3-S13.gz › rap/doc/phpdoc/syntax/RdfParser.html]

xml version="1.0" encoding="iso-8859-1"?


Docs For Class RdfParser


## Class RdfParser

Description

Description |
Vars (details)
| Methods (details)

An RDF paser.

This class reads RDF data from files or URIs and generates models out of it. All valid RDF XML syntaxes defined by the W3C in RDF/XML Syntax Specification (Revised)

- W3C Working Draft 10 October 2003

(http://www.w3.org/TR/2003/WD-rdf-syntax-grammar-20031010/) are supported. The parser is based on the PHP version of repat (http://phpxmlclasses.sourceforge.net/show\_doc.php?class=class\_rdf\_parser.html) by Luis Argerich (lrargerich@yahoo.com).

History:

- 12-06-2004 : improved namespace handling added (tobias.gauss@web.de)
- 08-13-2004 : Bug in rdf:resource="" fixed
- 08-10-2004 : function for converting strings to its unicode NFC form added. Benjamin Nowack <bnowack@appmosphere.com>
- 06-01-2004 : Bug in xml:lang fixed, some bugs in the handling of collections fixed
- 05-03-2004 : generateModel(): added optional parameter $rdfBaseURI to set the rdf base URI manually for strings when there's
  no xml:base stated <mail@d-westphal.de>.
- 11-18-2003 : Made compliant with the latest RDF Specification (10. October 2003) <radol@gmx.de>.
  Support for several aspects of the RDF Specification
  was added or fixed, among others:
  bugs in handling of xml:base and resolving relative URIs fixed,
  support for parseType="Literal" (XMLLiterals) added,
  support for parseType="Collection" added,
  handling of xml:lang improved,
  handling of containers improved,
  handling of empty property elements improved,
  handling of RDF vocabulary improved,
  some cases of reification fixed,
  Several methods have been changed, unused variables and
  parameters removed (see CVS for details).
  new private methods: \_is\_forbidden\_rdf\_property\_attribute(),
  \_is\_forbidden\_rdf\_property\_element ($local\_name),
  \_is\_rdf\_node\_element(),
  \_is\_forbidded\_node\_element(),
  \_report\_error(),
  \_handle\_collection\_element(),
  \_handle\_xml\_start\_element(),
  \_handle\_xml\_end\_element(),
  \_join\_name\_and\_declare\_prefix(),
  \_end\_collection(),
  \_start\_ns\_declaration\_handler(),
  removed private methods: \_delete\_elements(),
  \_end\_empty\_resource\_property(),
  rdf\_resolve\_uri()
  BUGS WHICH HAVE NOT BEED FIXED:
  - correct handling of non US-ASCII characters
- 07-27-2003 : Functions addapted to the new class tree (MemModel extends Model)
- 04-23-2003 : Bug concerning bnode recognition fixed.
- 04-03-2003 : Bug concerning bnode identifier generation fixed.
- 01-10-2003 : Support for rdf:datatype and rdf:nodeID added.
- 01-05-2003 : Support for rdf:seeAlso added.
- 11-07-2002 : Possibility to pass RDF code directly to the method generateModel() added.
- 10-25-2002 : Bug concerning 1 character long literals fixed.
- 09-24-2002 : Recognition of blank nodes improved.
- 09-10-2002 : First version of this class.

- access: public
- author: Luis Argerich <lrargerich@yahoo.com>, Chris Bizer <chris@bizer.de>, Radoslaw Oldakowski <radol@gmx.de> Daniel Westphal <mail@d-westphal.de>
- version: V0.9.1

Located in /syntax/RdfParser.php (line 79)

```
Object
   |
   --RdfParser
```

Variable Summary

Description |
Vars (details)
|
Methods (details)

mixed
$model

mixed
$rdf\_parser

Method Summary

Description |
Vars (details)
|
Methods (details)

object MemModel
&generateModel
(string $base, [boolean $rdfBaseURI = false], [mixed $model = false])

void
\_is\_forbidden\_rdf\_property\_attribute
(mixed $local\_name)

void
\_parse\_uri
(mixed $uri, mixed $buffer, mixed &$scheme, mixed &$authority, mixed &$path, mixed &$query, mixed &$fragment)

void
\_report\_error
(mixed $error)

Variables

Description |
Vars (details)
|
Methods (details)

mixed
$model
(line 82)

mixed
$rdf\_parser
(line 81)

Methods

Description |
Vars (details)
Methods (details)

generateModel (line 2254)

Generates a new MemModel from a URI, a file or from memory.

If you want to parse an RDF document, pass the URI or location in the filesystem of the RDF document. You can also pass RDF code direct to the function. If you pass RDF code directly to the parser and there is no xml:base included, you should set the base URI manually using the optional second parameter $rdfBaseURI. Make sure that here are proper namespace declarations in your input document.

- access: public

object MemModel

&generateModel
(string $base, [boolean $rdfBaseURI = false], [mixed $model = false])

- string
  $base
- boolean
  $rdfBaseURI

\_is\_forbidden\_rdf\_property\_attribute (line 353)

void

\_is\_forbidden\_rdf\_property\_attribute
(mixed $local\_name)

\_parse\_uri (line 520)

void

\_parse\_uri
(mixed $uri, mixed $buffer, mixed &$scheme, mixed &$authority, mixed &$path, mixed &$query, mixed &$fragment)

\_report\_error (line 1100)

void

\_report\_error
(mixed $error)

#### Inherited Methods


Inherited From Object

> Object::toString()

Documentation generated on Thu, 7 Jul 2005 13:42:56 +0200 by phpDocumentor 1.3.0RC3
